# The Association Between *STAT4* rs7574865 Polymorphism and the Susceptibility of Autoimmune Thyroid Disease: A Meta-Analysis

**DOI:** 10.3389/fgene.2018.00708

**Published:** 2019-01-07

**Authors:** Xueren Gao, Jianguo Wang, Yongguo Yu

**Affiliations:** Department of Pediatric Endocrinology and Genetics, Shanghai Institute for Pediatric Research, Xinhua Hospital, School of Medicine, Shanghai Jiao Tong University, Shanghai, China

**Keywords:** *STAT4*, polymorphism, susceptibility, autoimmune thyroid disease, meta-analysis

## Abstract

**Objectives:** The signal transducer and activator of transcription 4 (*STAT4*) gene encodes an important transcription factor that transmits signals induced by several cytokines associated with autoimmune diseases and has been identified as a susceptibility gene for numerous autoimmune disorders. The association between *STAT4* rs7574865 polymorphism and the susceptibility of autoimmune thyroid disease (AITD) has been investigated in previous case-control studies. However, the investigation results were inconsistent. Hence, a meta-analysis was performed to draw a more reliable conclusion about it.

**Methods:** All relevant studies were searched in Embase, PubMed, Web of Science, and China National Knowledge Infrastructure, till August 20, 2018. The pooled odds ratios (ORs) with 95% confidence intervals (CIs) were used to evaluate the strength of the association.

**Results:** A total of five independent case-control studies with 1707 AITD patients and 2316 controls were included in the present meta-analysis. The overall pooled analysis indicated that *STAT4* rs7574865 polymorphism was significantly associated with AITD susceptibility [TT vs. GG: OR = 1.63, 95%CI = 1.24–2.15, *P*_Z_ = 0.0005; TT vs. (TG+GG): OR = 1.55, 95%CI = 1.26–1.91, *P*_Z_ < 0.0001]. However, the subgroup analysis showed a significant association of *STAT4* rs7574865 polymorphism with AITD susceptibility in Asian population, but not in African population. *STAT4* rs7574865 polymorphism was significantly associated both with Graves’ disease (GD) and Hashimoto’s thyroiditis (HT) susceptibility.

**Conclusion:** This meta-analysis showed a significant association between *STAT4* rs7574865 polymorphism and AITD susceptibility. However, further studies with larger sample sizes and other ethnicities are still required to confirm the findings.

## Introduction

Autoimmune thyroid disease (AITD) affects approximately 5% of the general population (Tunbridge et al., 1977; Hollowell et al., 2002). Graves’ disease (GD) and Hashimoto’s thyroiditis (HT) are two main subtypes of AITD and mainly characterized by thyrotoxicosis and hypothyroidism, respectively (Brix and Hegedüs, 2012). Although GD and HT present different clinical phenotypes, both diseases share common immunopathogenic features, including lymphocytic infiltration of the thyroid and the production of thyroid autoantibodies (Weetman, 2001), suggesting that immune-modulating genes participate in the occurrence of AITD. During the last several decades, great progress has been made in the study of AITD risk factors. Epidemiological studies have pointed out several environmental factors triggering AITD, such as viral infections, drugs, irradiation and iodine intake, which probably involve interference with thyroid function, immune stimulation, direct toxic effects on thyrocytes, or other immunomodulatory effects (Orgiazzi, 2012; Antonelli et al., 2015). Furthermore, increasing evidences have suggested that individual genetic factors also play an important role in the risk of AITD (Ban et al., 2005; Durães et al., 2014; Lin et al., 2016). The exploration of genetic factors associated with AITD risk will contribute to risk assessment and prevention of disease.

Signal transducer and activator of transcription 4 (*STAT4*) gene, located on human chromosome 2q32.3, encodes a member of the STAT family of transcription factors. In response to cytokines and growth factors, STAT family members are phosphorylated by the receptor related kinases, and then form homo- or heterodimers that translocate to the cell nucleus in which they function as transcription activators (Wurster et al., 2000). This protein is responsible for mediating responses to interleukin-12 (IL12) in lymphocytes, and regulating the production of interferon-γ (IFN-γ) and the differentiation of T-helper type 1 (Th1) cells (Thierfelder et al., 1996; Kaplan, 2005; Korman et al., 2008). Therefore it is an important mediator of the immune response and it is highly plausible that any change in *STAT4* expression or activity can alter the function and response of the normal immune system, leading to immunosuppression or autoimmune disorders.

Single nucleotide polymorphism (SNP) was the most common type of genetic variations in human genome. Some potentially functional SNPs have been reported to be associated with the risk of human complex diseases, such as cancer, schizophrenia, stroke, AITD (Park et al., 2011; Gao et al., 2015; Fang et al., 2018; Shi et al., 2018; Zhang et al., 2018). Thereinto a SNP (rs7574865 G/T) located in the third intron of *STAT4* gene is worthy of being noted. Although it is not located in the promoter or 3^′^-untranslated region, it can influence *STAT4* expression. Compared to rs7574865 G allele, the presence of T allele can significantly enhance *STAT4* mRNA transcription and protein expression (Jiang et al., 2013; Lamana et al., 2015). Even though the molecular mechanism of the SNP is unclear, it has been linked to the susceptibility of numerous autoimmune diseases, such as systemic lupus erythematosus, rheumatoid arthritis and AITD (Kawasaki et al., 2008; Kobayashi et al., 2008; Gupta et al., 2018). For the association of *STAT4* rs7574865 polymorphism with AITD susceptibility, some inconsistent results were reported. For instance, some studies argued that rs7574865 polymorphism was significantly associated with AITD susceptibility, but others showed no significant association, which might be due to a small sample size in a single study (Ben Hamad et al., 2011; Park et al., 2011; Yan et al., 2014; Hiz et al., 2015; Zhao et al., 2016). In order to draw a more reliable conclusion, we performed a pooled analysis of data from all relevant studies.

## Materials and Methods

### Literature Retrieval Strategy

Embase, PubMed, Web of Science, and China National Knowledge Infrastructure (CNKI) electronic databases were searched for eligible studies published till August 20, 2018. The following terms were used for searching titles or abstracts: “*STAT4* OR Signal transducer and activator of transcription 4,” “polymorphism OR SNP OR variation” and “autoimmune thyroid disease OR AITD”. In addition, the original or review reports were screened carefully and searched manually for more eligible studies based on their references.

### Study Selection

Eligible studies were chosen by two authors independently (XR Gao and JG Wang) according to the following inclusion criteria: (i) case-control studies that assessed the relationship between *STAT4* rs7574865 polymorphism and AITD susceptibility; (ii) studies that contained the distribution of allelic or genotypic frequencies of *STAT4* rs7574865 polymorphism in all participants; (iii) studies that scored more than six stars based on the Newcastle-Ottawa Scale (NOS), which was adopted to assess the methodological quality of each candidate study (Stang, 2010). The NOS comprised three assessment categories, including comparability, exposure and selection, which contained one, three, and four items, respectively. In the selection and exposure categories, each eligible item was endowed with one star, and in the comparability category, eligible item was endowed with at most two stars.

### Data Extraction

Two investigators (XR Gao and JG Wang) independently extracted data from each eligible literature, including first author’s name, publication year, country of origin, ethnicity, genotyping method, the total number of participants, allelic or genotypic frequencies, and *P* value for Hardy-Weinberg equilibrium (HWE). Any controversial content would be resolved by discussing with a third author (YG Yu).

### Statistical Analysis

Hardy-Weinberg equilibrium status of the controls was measured by the goodness-of-fit chi-square test. *P*_HWE_ value > 0.05 indicated that the genotype frequency of the controls was consistent with HWE. Chi-square based Q statistic were used to evaluate heterogeneity between studies. If *P*_Q_ value < 0.1, the heterogeneity between studies was significant. Thus, the random-effects model would be adopted to calculate the pooled odds ratios (ORs), otherwise, the fixed-effects model would be chosen. The pooled ORs with 95% confidence intervals (CIs) were used to assess the strength of the association. The statistical significance of the strength was determined by Z-test. *P*_z_ value < 0.05 was considered statistically significant. A total of five genetic comparison models were used in the present analysis, including homozygote model (TT vs. GG), heterozygous model (TG vs. GG), dominant model [(TT+TG) vs. GG], recessive model [TT vs. (TG+GG)], and allele comparison model (T vs. G). In addition, the stability of the pooled results and the potential publication bias were evaluated by sensitivity analysis and funnel plots, respectively. All of the above analyses were performed by RevMan 5.0 software (The Cochrane Collaboration, Copenhagen).

## Results

### Characteristics of Studies

**FIGURE 1:**
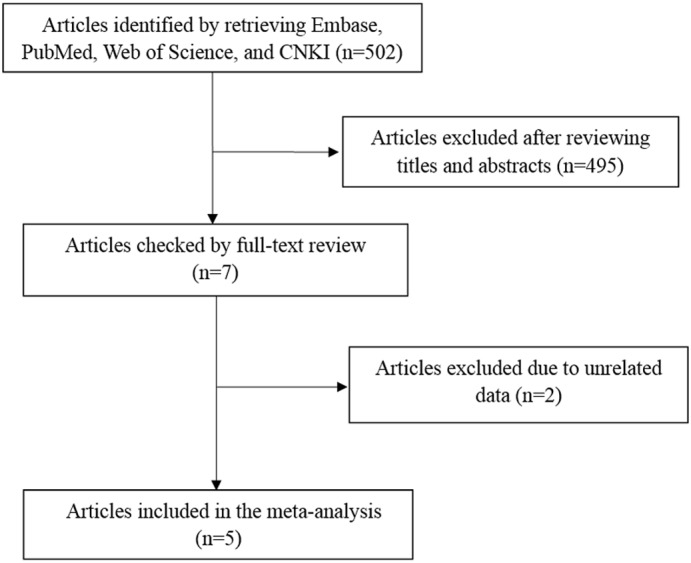
Flow diagram of literature retrieval and selection process.

A total of 502 articles were originally obtained by literature retrieval. According to our inclusion criteria, 5 articles published between the years 2011 and 2016 were finally included in the present meta-analysis (Figure 1). As shown in Table 1, there were 1707 AITD patients and 2316 controls that were used to assess the relationship between *STAT4* rs7574865 polymorphism and AITD susceptibility. Thereinto, 1548 AITD patients and 2116 controls belonged to Asian population. Other paticipants including 159 AITD patients and 200 controls belonged to African population. For the NOS, all the studies scored more than 6 stars, and were considered to be of high methodological quality (Supplementary Table S1).

**Table 1:** The main characteristics of the included studies.

Authors	Year	Country	Ethnicity	Genotyping methods	Cases					Controls				PHWE	NOS
					GG	GT	TT	Total	Type	GG	GT	TT	Total		
Zhao et al.	2016	China	Asian	Sequencing	26	15	20	61	GD	23	22	5	50	0.94	7 stars
Hiz et al.	2015	Turkey	Asian	real time PCR	13	2	0	15	AITD	59	39	5	103	0.65	8 stars
Yan et al.	2014	China	Asian	PCR-LDR	427	474	143	1044	AITD	408	404	91	903	0.54	8 stars
					287	304	99	690	GD						
					140	170	44	354	HT						
Park et al.	2011	Korea	Asian	TaqMan	367		61	428	AITD	954		106	1060	NA	8 stars
					208		33	241	GD						
					159		28	187	HT						
Ben Hamad et al.	2011	Tunisia	African	TaqMan	102	50	7	159	AITD	135	63	2	200	0.07	8 stars


*AITD, autoimmune thyroid disease; GD, Graves’ disease; HT, Hashimoto’s thyroiditis; NA, not available.*

### Quantitative Synthesis

The association of *STAT4* rs7574865 polymorphism with AITD susceptibility was shown in Table 2 and Supplementary Figure S1. The overall pooled analysis indicated that *STAT4* rs7574865 polymorphism was significantly associated with AITD susceptibility [TT vs. GG: OR = 1.63, 95%CI = 1.24–2.15, *P*_Z_ = 0.0005; TT vs. (TG+GG): OR = 1.55, 95%CI = 1.26–1.91, *P*_Z_ < 0.0001]. The subgroup analysis based on ethnicity showed a significant association of *STAT4* rs7574865 polymorphism with AITD susceptibility in Asian population [TT vs. GG: OR = 1.57, 95%CI = 1.18–2.08, *P*_Z_ = 0.002; TT vs. (TG+GG): OR = 1.51,95%CI = 1.23–1.87, *P*_Z_ = 0.0001], but not in African population. The stratified analysis by disease type showed that *STAT4* rs7574865 polymorphism was significantly associated both with GD [TT vs. (TG+GG): OR = 1.57, 95%CI = 1.24–2.00, *P*_Z_ = 0.0002] and HT susceptibility [TT vs. (TG+GG): OR = 1.39, 95%CI = 1.04–1.86, *P*_Z_ = 0.03].

**Table 2:** The association between *STAT4* rs7574865 polymorphism and AITD susceptibility.

Comparison model	Subgroup	No. of studies	Sample size (Cases/Controls)	*P*_H_	Effect model	OR (95% CI)	*P*_Z_
Homozygote comparison	AITD	4	738/728	0.20	Fixed	**1.63 [1.24, 2.15]**	**0.0005**
(TT vs. GG)	Asian	3	629/591	0.23	Fixed	**1.57 [1.18, 2.08]**	**0.002**
	African	1	109/137	–	–	4.63 [0.94, 22.77]	0.06
Heterozygote comparison	AITD	4	1109/1153	0.13	Fixed	1.05 [0.89, 1.25]	0.54
(TG vs. GG)	Asian	3	957/955	0.06	Random	0.71 [0.33, 1.52]	0.37
	African	1	152/198	–	–	1.05 [0.67, 1.65]	0.83
Dominant model	AITD	4	1279/1256	0.18	Fixed	1.15 [0.98, 1.35]	0.09
[(TT+TG) vs. GG]	Asian	3	1120/1056	0.08	Random	0.93 [0.49, 1.77]	0.83
	African	1	159/200	–	–	1.16 [0.75, 1.80]	0.51
Recessive model	AITD	5	1707/2316	0.18	Fixed	**1.55 [1.26, 1.91]**	**<0.0001**
[TT vs. (TG+GG)]	HT	2	541/1963	0.46	Fixed	**1.39 [1.04, 1.86]**	**0.03**
	GD	3	992/2013	0.14	Fixed	**1.57 [1.24, 2.00]**	**0.0002**
	Asian	4	1548/2116	0.22	Fixed	**1.51 [1.23, 1.87]**	**0.0001**
	African	1	159/200	–	–	4.56 [0.93, 22.26]	0.06
Allele comparison	AITD	4	2558/2512	0.08	Random	1.22 [0.89, 1.67]	0.23
(T vs. G)	Asian	3	2240/2112	0.04	Random	1.12 [0.62, 2.01]	0.72
	African	1	318/400	–	–	1.25 [0.86, 1.83]	0.25


*P_H_, P value for heterogeneity. Bold terms indicate significant OR.*

### Sensitivity Analysis and Publication Bias

**FIGURE 2:**
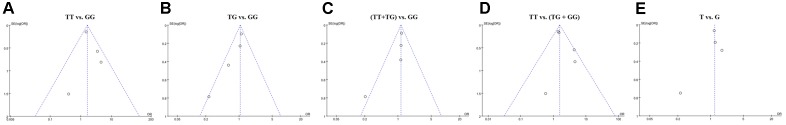
Funnel plot of the association of *STAT4* rs7574865 polymorphism with the susceptibility of autoimmune thyroid disease. **(A–E)** Indicates homozygote comparison, heterozygote comparison, dominant model, recessive model, allele comparison, respectively.

Sensitivity analysis was conducted by removing a single study at a time and reanalyzing the pooled results. The results of the analysis indicated that the omission of any one study could not substantially change the overall pooled results under homozygote comparison (TT vs. GG), heterozygous comparison (TG vs. GG), and recessive comparison [TT vs. (TG+GG)] models. However, under dominant comparison [(TT+TG) vs. GG] and allele comparison (T vs. G) models, significant changes were observed after removing Hiz’s study (Supplementary Figure S2). The results of the funnel plots did not show any significant asymmetry in the overall analysis, suggesting a lack of publication bias (Figure 2).

## Discussion

In recent years, the association of *STAT4* rs7574865 polymorphism with AITD susceptibility has attracted a lot of attention. Although this polymorphism is located in the non-coding region of *STAT4* gene, it can significantly affect *STAT4* expression and is frequently considered to be related to AITD susceptibility (Park et al., 2011; Yan et al., 2014; Hiz et al., 2015; Zhao et al., 2016). Zhao et al. (2016) analyzed distribution of the genotype and allele frequency of *STAT4* rs7574865 polymorphism in a Chinese Han Population of Shanxi including 61 GD patients and 50 healthy controls, and found that *STAT4* rs7574865 T allele significantly increased the risk of GD. Hiz et al. (2015) observed that *STAT4* rs7574865 T allele increased AITD susceptibility in a Turkish population containing 15 AITD cases and 103 controls. Yan et al. (2014) also investigated the role of the *STAT4* rs7574865 polymorphism in AITD susceptibility by a Chinese case-control study (1044 patients affected with AITD and 903 healthy controls), and the results indicated that the frequencies of *STAT4* rs7574865 genotypes in GD patients were significantly different from that in the controls, and the T allele frequency of GD patients was also significantly higher than the controls. Park et al. (2011) found that compared with individuals carrying GT and GG gentypes, individuals carrying TT gentypes had an incresed risk of AITD in Korean samples of 428 AITD and 1060 controls. All of the above results suggested that *STAT4* rs7574865 polymorphism could act as a genetic risk factor of AITD. However, a case-control study (159 patients affected with AITD and 200 healthy controls) based on Tunisian population showed no significant association of *STAT4* rs7574865 polymorphism with the risk of AITD (Ben Hamad et al., 2011). Thus, it is urgent for us to take effective measures to get robust conclusions. Meta-analysis is a very powerful tool for analyzing cumulative data of studies where the sample sizes are small and the statistical power is low. In the present study, we adopted the scientific method to assess the association of *STAT4* rs7574865 polymorphism with AITD susceptibility, and observed that *STAT4* rs7574865 polymorphism was significantly associated with AITD susceptibility. In addition, the subgroup analysis based on ethnicity showed a significant association of *STAT4* rs7574865 polymorphism with AITD susceptibility in Asian population, but not in African population. The stratified analysis based on disease type indicated that *STAT4* rs7574865 polymorphism was significantly related to the susceptibility of both GD and HT.

The present results provided a stronger evidence for the association between *STAT4* rs7574865 polymorphism and AITD susceptibility, but some existing limitations should not be ignored. Firstly, due to a limited number of related studies, we could not assess the association of rs7574865 polymorphism with AITD susceptibility among other ethnicities, such as Caucasians. Secondly, the sample size in the study was small, which reduced the clinical impact of the results. Therefore, further studies with larger sample sizes and other ethnicities are still required to confirm the current findings. Last but not least, our meta-analysis was based on an unadjusted assessment. A more precise analysis should be carried out if personal data such as gender, lifestyle and environmental exposure factors were available.

## Conclusion

This meta-analysis showed a significant association between *STAT4* rs7574865 polymorphism and AITD susceptibility, especially in Asian population. The polymorphism may be used as a predictive marker for AITD predisposition.

## Author Contributions

YY and XG designed the study. XG and JW searched the databases, collected the full-text papers, extracted and analyzed the data, and wrote the manuscript. YY reviewed the manuscript.

## Conflict of Interest Statement

The authors declare that the research was conducted in the absence of any commercial or financial relationships that could be construed as a potential conflict of interest.

## Supplementary Material

FIGURE S1 Forest plots of the association of *STAT4* rs7574865 polymorphism with the susceptibility of autoimmune thyroid disease.

FIGURE S2 Forest plots of the association of *STAT4* rs7574865 polymorphism with the susceptibility of autoimmune thyroid disease after removing Hiz’s study.

TABLE S1 The quality assessment of all included studies based on the Newcastle-Ottawa Scale.

## References

[B1] Antonelli A. Ferrari S. M. Corrado A. Di Domenicantonio A. Fallahi P. (2015). Autoimmune thyroid disorders. *Autoimmun. Rev.* 14 174–180. 10.1016/j.autrev.2014.10.016 25461470

[B2] Ban Y. Tozaki T. Taniyama M. Tomita M. Ban Y. (2005). Association of a CTLA-4 3^′^ untranslated region (CT60) single nucleotide polymorphism with autoimmune thyroid disease in the Japanese population. *Autoimmunity* 38 151–153. 10.1080/08916930500050319 16040335

[B3] Ben Hamad M. Cornelis F. Mbarek H. Chabchoub G. Marzouk S. Bahloul Z. (2011). Signal transducer and activator of transcription and the risk of rheumatoid arthritis and thyroid autoimmune disorders. *Clin. Exp. Rheumatol.* 29 269–274. 21418779

[B4] Brix T. H. Hegedüs L. (2012). Twin studies as a model for exploring the aetiology of autoimmune thyroid disease. *Clin. Endocrinol.* 76 457–464. 10.1111/j.1365-2265.2011.04318.x 22168537

[B5] Durães C. Moreira C. S. Alvelos I. Mendes A. Santos L. R. Machado J. C. (2014). Polymorphisms in the TNFA and IL6 genes represent risk factors for autoimmune thyroid disease. *PLoS One* 9:e105492. 10.1371/journal.pone.0105492 25127106 PMC4134306

[B6] Fang M. Huang W. Mo D. Zhao W. Huang R. (2018). Association of five snps in cytotoxic T-lymphocyte antigen 4 and cancer susceptibility: evidence from 67 studies. *Cell Physiol. Biochem.* 47 414–427. 10.1159/000489953 29794444

[B7] Gao X. R. Zhang S. L. Zhu Z. Z. (2015). Lysyl oxidase rs1800449 polymorphism and cancer risk among Asians: evidence from a meta-analysis and a case-control study of colorectal cancer. *Mol. Genet. Genomics* 290 23–28. 10.1007/s00438-014-0896-3 25112403

[B8] Gupta V. Kumar S. Pratap A. Singh R. Kumari R. Kumar S. (2018). Association of ITGAM, TNFSF4, TNFAIP3 and STAT4 gene polymorphisms with risk of systemic lupus erythematosus in a North Indian population. *Lupus* 27 1973–1979. 10.1177/0961203318786432 30041578

[B9] Hiz M. M. Kılıç S. Işık S. Ogretmen Z. Silan F. (2015). Contribution of the STAT4 rs7574865 gene polymorphism to the susceptibility to autoimmune thyroiditis in healthy Turk population and psoriatic subgroups. *Cent. Eur. J. Immunol.* 40 437–441. 10.5114/ceji.2015.57146 26862307 PMC4737749

[B10] Hollowell J. G. Staehling N. W. Flanders W. D. Hannon W. H. Gunter E. W. Spencer C. A. (2002). Serum TSH, T(4), and thyroid antibodies in the United States population (1988 to 1994): National Health and Nutrition Examination Survey (NHANES III). *J. Clin. Endocrinol. Metab.* 87 489–499. 10.1210/jcem.87.2.8182 11836274

[B11] Jiang D. K. Sun J. Cao G. Liu Y. Lin D. Gao Y. Z. (2013). Genetic variants in STAT4 and HLA-DQ genes confer risk of hepatitis B virus-related hepatocellular carcinoma. *Nat. Genet.* 45 72–75. 10.1038/ng.2483 23242368 PMC4105840

[B12] Kaplan M. H. (2005). STAT4: a critical regulator of inflammation in vivo. *Immunol. Res.* 31 231–242. 10.1385/IR:31:3:231 15888914

[B13] Kawasaki A. Ito I. Hikami K. Ohashi J. Hayashi T. Goto D. (2008). Role of STAT4 polymorphisms in systemic lupus erythematosus in a Japanese population: a case-control association study of the STAT1-STAT4 region. *Arthritis Res. Ther.* 10:R113. 10.1186/ar2516 18803832 PMC2592800

[B14] Kobayashi S. Ikari K. Kaneko H. Kochi Y. Yamamoto K. Shimane K. (2008). Association of STAT4 with susceptibility to rheumatoid arthritis and systemic lupus erythematosus in the Japanese population. *Arthritis Rheum.* 58 1940–1946. 10.1002/art.23494 18576330

[B15] Korman B. D. Kastner D. L. Gregersen P. K. Remmers E. F. (2008). STAT4: genetics, mechanisms, and implications for autoimmunity. *Curr. Allergy Asthma Rep.* 8 398–403. 10.1007/s11882-008-0077-8 18682104 PMC2562257

[B16] Lamana A. López-Santalla M. Castillo-González R. Ortiz A. M. Martín J. García-Vicuña R. (2015). The minor allele of rs7574865 in the STAT4 gene is associated with increased mrna and protein expression. *PLoS One* 10:e0142683. 10.1371/journal.pone.0142683 26569609 PMC4646635

[B17] Lin J. D. Yang S. F. Wang Y. H. Fang W. F. Lin Y. C. Lin Y. F. (2016). Analysis of associations of human BAFF gene polymorphisms with autoimmune thyroid diseases. *PLoS One* 11:e0154436. 10.1371/journal.pone.0154436 27136204 PMC4852922

[B18] Orgiazzi J. (2012). Thyroid autoimmunity. *Presse Med.* 41 e611–e625. 10.1016/j.lpm.2012.10.002 23164679

[B19] Park Y. Lee H. S. Park Y. Min D. Yang S. Kim D. (2011). Evidence for the role of STAT4 as a general autoimmunity locus in the korean population. *Diabetes Metab. Res. Rev.* 27 867–871. 10.1002/dmrr.1263 22069275

[B20] Shi J. He W. Wang Y. Hua J. (2018). Tagging functional polymorphism in 3’ untranslated region of methylene tetrahydrofolate reductase and risk of ischemic stroke. *Cell Physiol. Biochem.* 46 1019–1026. 10.1159/000488833 29669328

[B21] Stang A. (2010). Critical evaluation of the newcastle-ottawa scale for the assessment of the quality of nonrandomized studies in meta-analyses. *Eur. J. Epidemiol.* 25 603–605. 10.1007/s10654-010-9491-z 20652370

[B22] Thierfelder W. E. van Deursen J. M. Yamamoto K. Tripp R. A. Sarawar S. R. Carson R. T. (1996). Requirement for Stat4 in interleukin-12-mediated responses of natural killer and T cells. *Nature* 382 171–174. 10.1038/382171a0 8700208

[B23] Tunbridge W. M. Evered D. C. Hall R. Appleton D. Brewis M. Clark F. (1977). The spectrum of thyroid disease in a community: the whickham survey. *Clin. Endocrinol.* 7 481–493. 10.1111/j.1365-2265.1977.tb01340.x 598014

[B24] Weetman A. P. (2001). Determinants of autoimmune thyroid disease. *Nat. Immunol.* 2 769–770. 10.1038/ni0901-769 11526381

[B25] Wurster A. L. Tanaka T. Grusby M. J. (2000). The biology of Stat4 and Stat6. *Oncogene* 19 2577–2584. 10.1038/sj.onc.1203485 10851056

[B26] Yan N. Meng S. Zhou J. Xu J. Muhali F. S. Jiang W. (2014). Association between STAT4 gene polymorphisms and autoimmune thyroid diseases in a Chinese population. *Int. J. Mol. Sci.* 15 12280–12293. 10.3390/ijms150712280 25019342 PMC4139844

[B27] Zhang S. Zhou N. Liu R. Rao W. Yang M. Cao B. (2018). Association between polymorphisms of the complement 3 gene and schizophrenia in a han Chinese population. *Cell Physiol. Biochem.* 46 2480–2486. 10.1159/000489654 29742493

[B28] Zhao J. Y. Liang W. F. He M. F. Wang F. Lan L. Z. (2016). Research on correlation between the graves disease in the han chinese of shanxi area. *China Foreign Med. Treat.* 4 54–56.

