# Supplementary figures and images for: The Association Between STAT4 rs7574865 Polymorphism and the Susceptibility of Autoimmune Thyroid Disease: A Meta-Analysis
Source: Front Genet. 2019 Jan 7;9:708. doi: 10.3389/fgene.2018.00708 (PMC6330290; doi:10.3389/fgene.2018.00708)

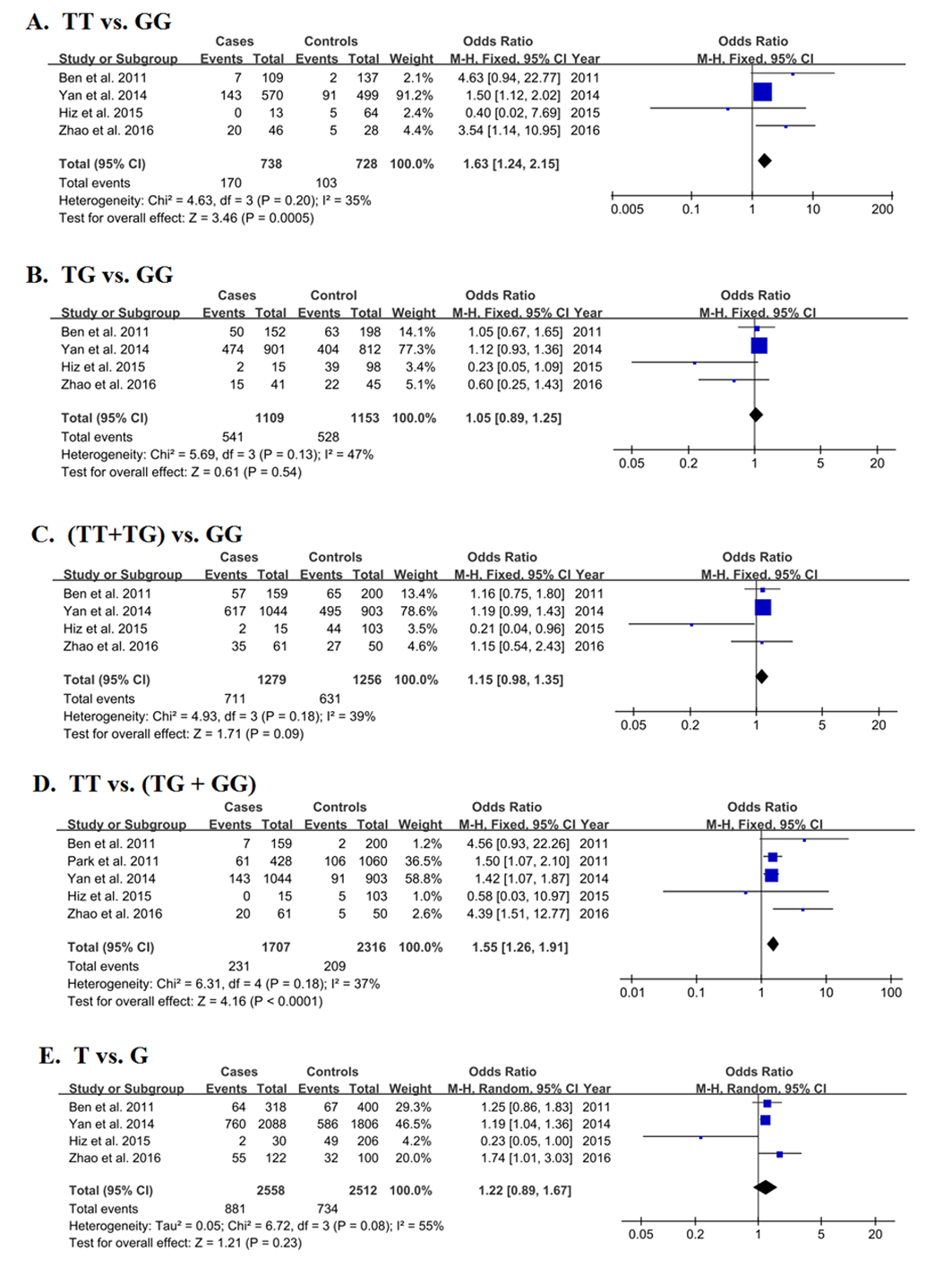

Supplement: FIGURE S1 — Forest plots of the association of STAT4 rs7574865 polymorphism with the susceptibility of autoimmune thyroid disease. [file Image_1.TIF]

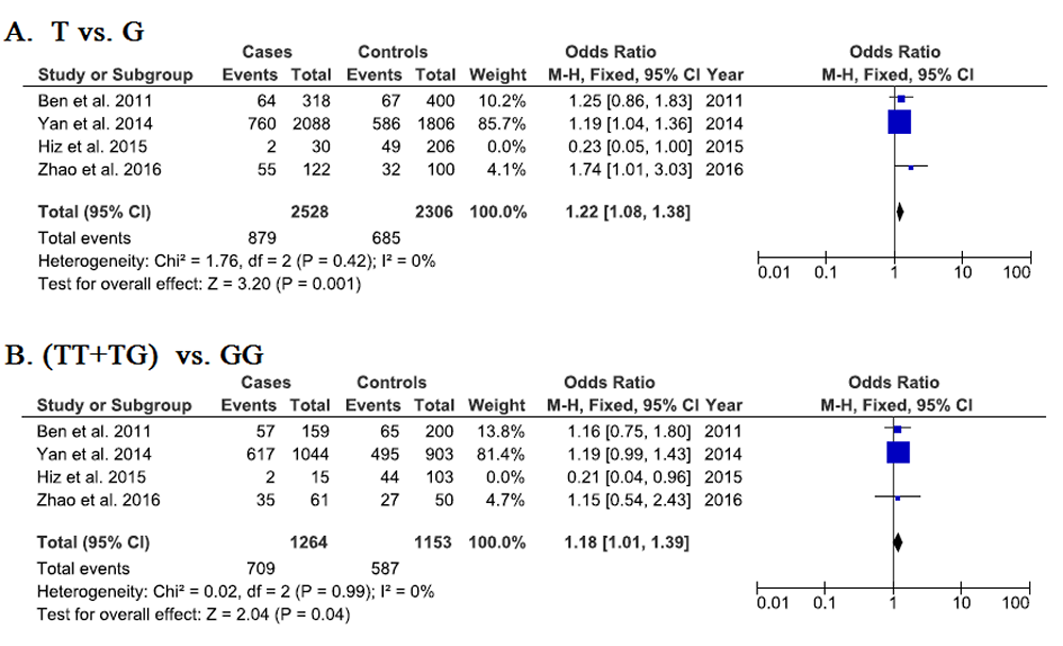

Supplement: FIGURE S2 — Forest plots of the association of STAT4 rs7574865 polymorphism with the susceptibility of autoimmune thyroid disease after removing Hiz’s study. [file Image_2.TIF]
