# Supplementary material for: The Association Between STAT4 rs7574865 Polymorphism and the Susceptibility of Autoimmune Thyroid Disease: A Meta-Analysis
Source: Front Genet. 2019 Jan 7;9:708. doi: 10.3389/fgene.2018.00708 (PMC6330290; doi:10.3389/fgene.2018.00708)
Supplement: TABLE S1 — The quality assessment of all included studies based on the Newcastle-Ottawa Scale. [file Table_1.DOCX]

Table S1. The quality assessment of all included studies based on the Newcastle-Ottawa Scale.

| Categories | Items | Zhao's study | Hiz's study | Yan's study | Park's study | Ben's study |
| --- | --- | --- | --- | --- | --- | --- |
| Selection | Adequacy of case definition | * | * | * | * | * |
|  | Representativeness of the cases | - | * | * | * | * |
|  | Selection of controls | * | * | * | * | * |
|  | Definition of controls | * | * | * | * | * |
| Comparability | Comparability of cases/controls | * | * | * | * | * |
| Exposure | Ascertainment of exposure | * | * | * | * | * |
|  | Same method of ascertainment for cases and controls | * | * | * | * | * |
|  | Non-Response rate | * | * | * | * | * |
